# Three-membered ring formation catalyzed by α-ketoglutarate-dependent nonheme iron enzymes

**DOI:** 10.1007/s11418-023-01760-4

**Published:** 2023-11-19

**Authors:** Richiro Ushimaru

**Affiliations:** 1 Graduate School of Pharmaceutical Sciences, The University of Tokyo, Tokyo, 113-0033 Japan; 2 Collaborative Research Institute for Innovative Microbiology, The University of Tokyo, Tokyo, 113-8657 Japan

**Keywords:** Cyclization, Natural product biosynthesis, Nonheme iron enzyme

## Abstract

Epoxides, aziridines, and cyclopropanes are found in various medicinal natural products, including polyketides, terpenes, peptides, and alkaloids. Many classes of biosynthetic enzymes are involved in constructing these ring structures during their biosynthesis. This review summarizes our current knowledge regarding how α-ketoglutarate-dependent nonheme iron enzymes catalyze the formation of epoxides, aziridines, and cyclopropanes in nature, with a focus on enzyme mechanisms.

## Introduction

Three-membered rings, such as epoxides, aziridines, and cyclopropanes, are ubiquitously found in natural products derived from various organisms, including plants and microorganisms [1]. These natural products often exhibit unique biological activities due to the distinctive chemical properties of the three-membered rings. The inherent large ring strain of these small cycles often accounts for their bioactivities via alkylation reactions when they interact with target molecules. For example, the epoxide-containing natural product fosfomycin serves as an inhibitor of MurA, which is involved in peptidoglycan synthesis, thereby exhibiting a potent antibiotic activity [2–4]. This inhibition results from the covalent bonding between fosfomycin and a cystine residue in MurA, triggered by the ring opening of the epoxide moiety [4]. The antitumor aziridine natural product mitomycin C is recognized for its ability to crosslink with DNA at specific sequences, forming covalent bonds following the opening of its aziridine ring [5–7]. Furthermore, the unique biological activities may also result from the distinct geometric properties of three-membered rings, such as the coplanarity of their three atoms and the rigidity of their ring conformation [8].

Due to the valuable medicinal properties and pharmaceutical applications of natural products containing three-membered rings, their biosynthetic mechanisms have garnered significant attention [1, 9, 10]. These small rings can be constructed through diverse enzymatic mechanisms, such as intramolecular nucleophilic substitution [11–13], atom transfer to alkenes [14], and cation-mediated cyclization [15–17]. Recent studies revealed that certain α-ketoglutarate (αKG)-dependent nonheme iron enzymes are also capable of catalyzing oxidative cyclization reactions to produce epoxides, aziridines, and cyclopropanes from the corresponding linear substrates, during the biosynthesis of various classes of natural products. Other αKG-dependent nonheme iron enzymes catalyze a diverse array of oxidative transformations, including hydroxylation, halogenation, and rearrangement reactions by cleaving a C–H bond in the substrate [18–23]. Although the mechanisms of the three-membered ring-forming iron enzymes are only partially understood, their reactions also appear to be initiated by H abstraction from unactivated carbon centers, in sharp contrast to the aforementioned, well-known cyclization mechanisms [18–20]. Since the highly strained rings are generally difficult to synthesize in a chemo- and stereo-selective manner by chemical methods, understanding the mechanisms of the nonheme iron cyclases will provide new opportunities to develop biocatalysts to access three-membered rings directly from the corresponding linear materials, via C–H bond activation. This review summarizes our understanding of the enzymatic chemistry of αKG-dependent nonheme iron enzymes involved in the natural production of epoxides, aziridines, and cyclopropanes, with a primary focus on enzyme mechanisms.

## Epoxidation

**Fig. 1:**
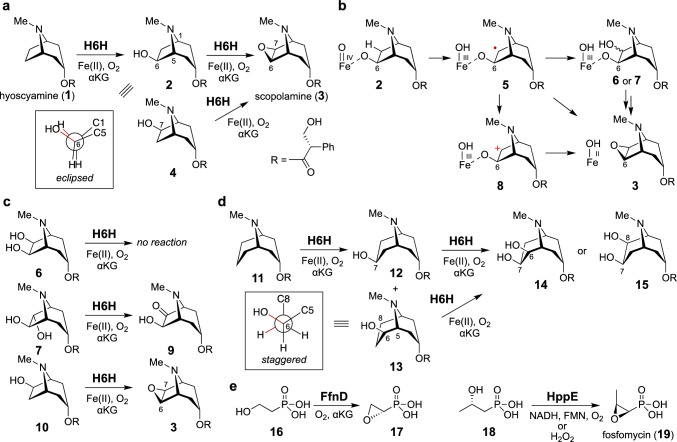
**a** H6H-catalyzed epoxidation of hyoscyamine (**1**). **b** Possible mechanisms for the H6H-catalyzed epoxidation. **c** Reactions of substrate analogues. **d** H6H-catalyzed oxidation of substrate analogues containing a 9-azabicyclo[3.3.1]nonane core. **e** Epoxidation reactions catalyzed by FfnD and HppE

Scopolamine (**3**) is a naturally occurring tropane alkaloid produced primarily by plants from the Solanaceae family, such as *Datura*, *Hyoscyamus*, and *Atropa* species (Fig. 1a) [24–26]. It is employed clinically for motion sickness and nausea prevention, as well as for managing conditions involving anxiety and agitation [24–26]. The molecular structure of scopolamine (**3**) is characterized by an 8-azabicyclo [3.2.1]octane core, derived from ornithine and two molecules of malonyl-CoA [27]. The αKG-dependent nonheme iron enzyme H6H catalyzes the final two steps of scopolamine biosynthesis from hyoscyamine (**1**) by installing the epoxide ring (Fig. 1a) [28–35]. The enzyme first introduces a hydroxyl group at C6 of hyoscyamine (**1**), as typically seen for this αKG-dependent nonheme iron enzyme family. The conversion of the hydroxylated intermediate **2** to scopolamine (**3**) was initially thought to involve 6,7-desaturated hyoscyamine, because it could be converted to scopolamine (**3**) in *Datura ferox* L [28]. The hypothetical biosynthetic intermediate, 6,7-desaturated hyoscyamine, is also accepted by H6H in vitro to produce scopolamine (**3**) [30]. However, a feeding experiment with [6-^18^O]6β-hydroxyhyoscyamine and *Datura scions* demonstrated that the transformation of 6β-hydroxyhyoscyamine (**2**) to scopolamine (**3**) does not involve a dehydration step [31]. This result was also consistent with the retention of the ^18^O atom when [6-^18^O]6β-hydroxyhyoscyamine was incubated with purified H6H [31]. The in vitro reaction of [7β-^2^H]6β-hydroxyhyoscyamine with H6H revealed the loss of the deuterium atom, indicating that the epoxidation reaction proceeds with the retention of the C7 stereoconfiguration [32].

Possible mechanisms of the epoxidation reaction catalyzed by H6H are depicted in Fig. 1b [36]. Since 6β-hydroxylation is the first step, the hydroxyl group in **2** may be located near the iron center in the H6H active site. Therefore, the iron center may be ligated by the C6-hydroxyl group in 6β-hydroxyhyoscyamine (**2**), similar to the proposed mechanism for the epoxidation reaction catalyzed by the αKG-independent nonheme iron enzyme HppE (Fig. 1e), although no evidence has been provided to prove this hypothesis. As a member of the αKG-dependent nonheme iron oxygenase family, an Fe(IV)-oxo species may be formed to abstract the H atom from the *exo*-C7 position in 6β-hydroxyhyoscyamine (**2**). The resulting radical intermediate **5** may react with the Fe(III)–OH to produce a 6,7-dihydroxylhyoscyamine intermediate (**6** or** 7**), which might undergo ring closure via intramolecular substitution. Alternatively, a carbocation species **8** may be formed via single electron transfer from the radical intermediate **5** to the Fe(III)–OH complex.

To explore the potential involvement of the diol species, two possible isomers **6** and** 7** were synthesized and subjected to testing with H6H (Fig. 1c) [36]. Only **7** was accepted by H6H, leading to the 7-keto product **9**, suggesting that the cyclization mechanism involving a diol intermediate (**6** or** 7**) is unlikely. Since H6H does not catalyze the hydroxylation of 6β-hydroxyhyoscyamine (**2**), it would be intriguing to determine what controls hydroxylation versus cyclization. It was hypothesized that the hydroxyl group adjacent to the site of H atom abstraction affects the course of the reaction. Consistent with this hypothesis, the synthetic 7β-hydroxylated compound (**10**) was transformed to scopolamine (**3**) by H6H, without producing the C6-hydroxylated product (Fig. 1c). In contrast, alcohol compounds containing a 9-azabicyclo[3.3.1]nonane core such as **12** and **13** were hydroxylated to generate the corresponding vicinal diols **14** and **15** when they were incubated with H6H (Fig. 1d). Thus, the ring size of the substrate (8-azabicyclo[3.2.1]octane versus 9-azabicyclo[3.3.1]nonane) appeared to correlate with the outcomes of the oxidation reactions by H6H. The small H–C6–C7–OH dihedral angle of 6β-hydroxyhyoscyamine (**2**) may be critical for the epoxidation activity. However, DFT calculations of radical intermediates derived from 6β-hydroxyhyoscyamine (**2**) and **13** revealed that the angle between the *p* orbital at C7 and the O–C6 bond would be small in both cases (less than 22 degrees). Thus, H6H may maintain the initial substrate geometry during the oxidation reactions. Another possible explanation is that a discrete radical species is only produced from a substrate with the staggered conformation (such as **12** and **13**), and that H atom abstraction and C–O bond formation occur in a concerted manner during the cyclization of 6β-hydroxyhyoscyamine (**2**) to scopolamine (**3**) [36].

H6H abstracts an H atom from C6 for the first hydroxylation reaction, but activates C7 during the second epoxidation reaction. C6 may also be the preferred site of H atom abstraction during the epoxidation. Kinetic analyses of the H6H-catalyzed hydroxylation and epoxidation were conducted using deuterated substrates [37]. Interestingly, during hydroxylation, the regioselectivity of H abstraction from C6 versus C7 was approximately 85:1, but it was reversed to be approximately 1:16, indicating a more than thousand-fold difference. The significant change of the regioselectivity of H abstraction could be explain by the proposed direct coordination of the hydroxyl group in **2** during the cyclization reaction.

**Fig. 2:**
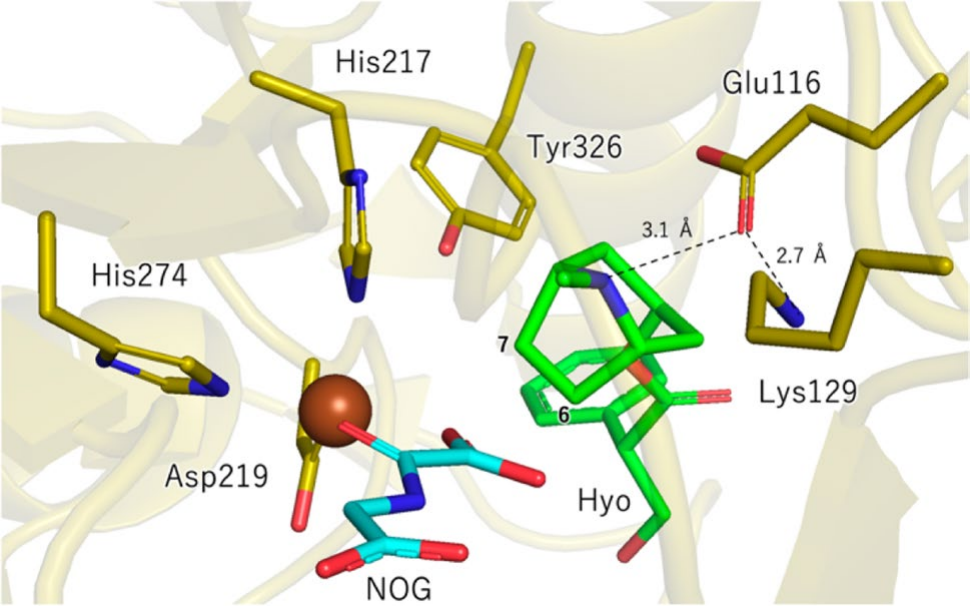
Crystal structure of H6H from *Datura metel* in complex with hyoscyamine and NOG. Hyo = hyoscyamine (**1**) (PDB 6TTN) [38]

The regioselectivity of H abstraction during the hydroxylation step was also investigated, based on crystal structures and QM/MM calculations [38]. The X-ray crystal structure of H6H from *Datura metel* in complex with hyoscyamine and *N*-oxalylglycine (NOG) revealed that C6 in hyoscyamine (**1**) is located 4.8 Å from the metal center, while C7 is positioned at a distance of 4.5 Å (Fig. 2). However, based on the QM/MM calculations with a model including the Fe(IV)-oxo species, H atom abstraction from C6 is significantly favored. Detailed geometrical analysis indicated that the Fe–O–H angle in the transition state was 141°, presumably enabling the energetically preferred σ-channel H abstraction. In contrast, the angle was 110° in the transition state structure for the H abstraction from C7, implying that this step follows the energetically less favored π-channel pathway. In silico amino acid substitutions in H6H, followed by energy calculations, were also conducted to identity the active site residues that might be critical in determining the regioselectivity of H abstraction. The results suggested that Tyr326 stabilizes the transition state of C6–H abstraction and destabilizes that of C7–H abstraction by interacting with the phenyl ring of the tropate side chain of hyoscyamine (**1**). The Lys129 residue is also important for the regioselectivity because it fixes the position of hyoscyamine (**1**) in the active site through electrostatic interactions with Glu116 that forms a hydrogen bond with the ammonium moiety in the substrate (Fig. 2) [38].

FfnD is a recently characterized cyclase that produces **17** from **16** during the biosynthesis of fosfonochlorin (Fig. 1e) [39]. In vitro enzyme assays with deuterated substrates revealed that the FfnD reaction retains the stereoconfiguration of the substrate. It is worth noting that this cyclization reaction bears similarities to the epoxidation reaction (from **18** to **19**) catalyzed by HppE in fosfomycin biosynthesis [40, 41]. However, there are several fundamental distinctions between these two enzymes. FfnD requires αKG and O_2_ as co-substrates, whereas HppE utilizes either the combination of NADH/FMN/O_2_ or H_2_O_2_. In addition, HppE epoxidation results in an inversion of the configuration. Consequently, the mechanism employed by FfnD may differ from that of HppE [42].

## Aziridination

**Fig. 3:**
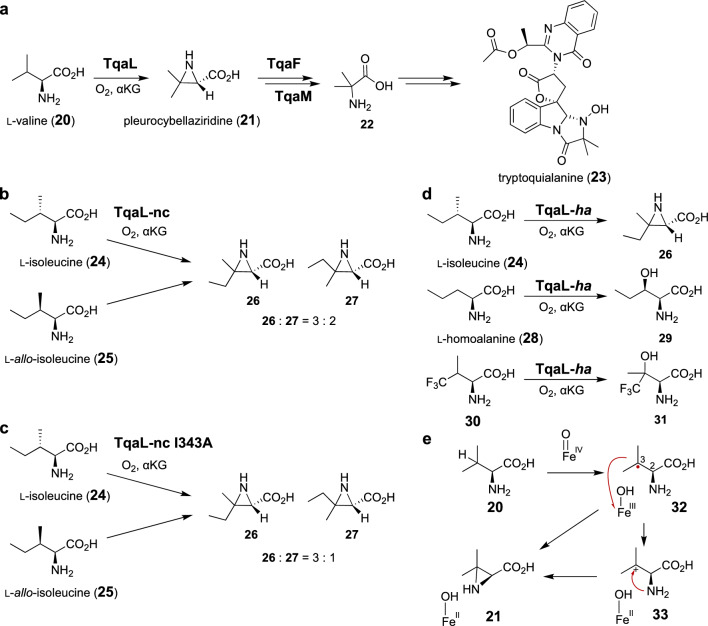
**a** Aziridination reaction catalyzed by TqaL. **b** TqaL-nc-catalyzed reactions of l-isoleucine and l-*allo*-isoleucine. **c** Reactions of the I343A variant of TaqL-nc. **d** Reaction of TqaL-*ha*. **e** Possible mechanism of the aziridination reaction

Tryptoquialanine (**23**) is a peptide-based natural product produced by *Penicillium aethiopicum* (Fig. 3a) [43, 44]. Biosynthetic studies revealed that tryptoquialanine (**23**) is assembled by the non-ribosomal peptide synthase (NRPS) TqaA, using amino acid components such as l-alanine, anthranilic acid, l-tryptophan, and 2-aminoisobutyric aid (**22**) [45–47]. 2-Aminoisobutyric aid (**22**) is a nonproteinogenic amino acid that is also present in other various biologically active fungal natural compounds, including alamethicin [48–50], zervamicin [51, 52], and emericellipsin [53]. Gene deletion in *P. aethiopicum* and in vitro enzyme characterization demonstrated that three sequential enzymatic reactions by TqaL, TqaF, and TqaM convert l-valine to 2-aminoisobutyric acid (**22**) [47, 54]. TqaL is an αKG-dependent nonheme iron oxygenase that catalyzes the aziridination of l-valine (**20**) to pleurocybellaziridine (**21**) (Fig. 3a) [54]. An in vitro analysis of stereoselectively deuterated l-valine (**20**) suggested that the aziridination reaction catalyzed by TqaL is not stereospecific, and instead involves both C3 retention and inversion of stereochemical courses [55]. A possible reaction mechanism is shown in Fig. 3e. The aziridination may be initiated by H atom abstraction from C3 of l-valine by an Fe(IV)-oxo species (**20**). The radical species **32** may be cyclized via a radical-based mechanism. Alternatively, **32** could be oxidized by the Fe(III)–OH species to generate the carbocation intermediate **33** that undergoes C–N bond-forming ring closure via a polar mechanism. The C2–C3 bond may undergo rapid rotation at the radical intermediate (**32**) stage [55].

**Fig. 4:**
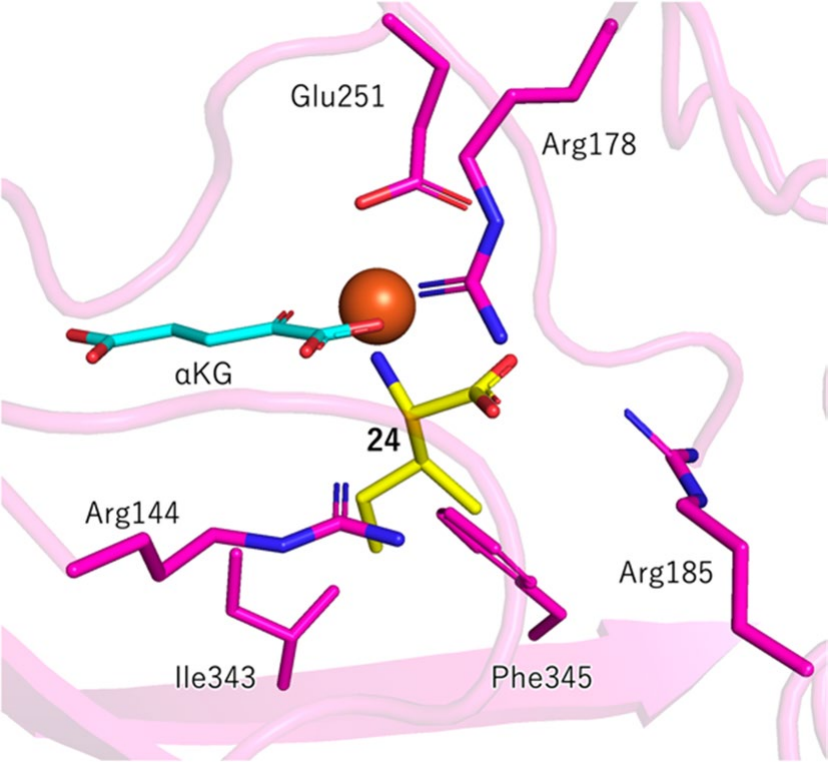
Predicted structure of TqaL-nc docked with l-isoleucine (**24**) [55]

TqaL from *Neurospora crassa* (TqaL-nc) can also accept l-isoleucine (**24**) and l-*allo*-isoleucine (**25**), in addition to the native substrate l-valine (**20**) (Fig. 3b) [55]. These two non-native substrates were converted to the same diastereomeric pairs of the aziridine product with a constant ratio (**26**: **27** = 3: 2), implying the involvement of an enzyme-controlled stereoconvergent process. The molecular basis of substrate binding was studied, based on a crystal structure analysis and structure prediction by Alphafold2 (Fig. 4) [55]. Accordingly, it was proposed that the amino group of the substrate interacts with Glu251, while the carboxylate moiety forms an H bonding network with three arginine residues, Arg144, Arg178, and Arg185. Notably, Ile343 and Phe345 are in the proximity of the side chain of the substrate, raising the hypothesis that these two residues may control the stereoselectivity of the aziridination of l-isoleucine (**24**) and l-*allo*-isoleucine (**25**). Consistent with this hypothesis, individual substitutions of Ile343 and Phe345 to alanine improved the stereoselectivity of the aziridination (**26**: **27** = 3: 1) (Fig. 3c). These studies demonstrated that the stereoselectivity could be rationally controlled by structure-based protein engineering, by dictating the conformation of the unstable reaction intermediate.

Several peptide natural products, such as atroviridins [56] and efrapeptins [57, 58] from fungal origins, contain isovaline moieties as an amino acid building block, implying that it is derived from l-isoleucine (**24**) via pathways analogous to that of 2-aminoisobutyric acid (**22**) in tryptoquialanine (**23**). A recent study indeed identified a homologue of TqaL (TqaL-*ha*) from the atroviridin-producing species *Hypocrea atroviridis*, which accepts l-isoleucine (**24**) to selectively produce **26** (Fig. 3d) [59]. Analysis of the TqaL-*ha* reaction by stopped-flow absorption spectroscopy using deuterated l-isoleucine (**24**) indicated that the Fe(IV)-oxo species is generated to abstract an H atom from the substrate. The results also suggested that a hydroxylated species is unlikely to be a reaction intermediate, because two isomers of β-hydroxylated isoleucines were not converted to the aziridine product **26** when incubated with TqaL-*ha*. Interestingly, when l-homoalanine (**28**) was employed as a possible substrate of TqaL-*ha*, the hydroxylated product **29** was produced without the formation of the aziridine product (Fig. 3d) [59]. This observed change of the reaction flux from aziridination to hydroxylation may be correlated with the stability of the cation intermediate. In the native reaction of **23** for TqaL-nc or **24** for TqaL-*ha*, following H abstraction by the Fe(IV)-oxo species, the radical intermediate may be oxidized to the tertiary carbocation via single electron transfer. In contrast, the corresponding radical intermediate derived from l-homoalanine (**28**) may undergo hydroxyl rebound with the Fe(III)-OH intermediate, because the hypothetical secondary carbocation that could have resulted from l-homoalanine (**28**) may be difficult to form, due to its decreased stability. The aziridination mechanism involving the carbocation intermediate was further supported by an experiment with 4-trifluorovaline (**30**), where the hydroxylated product **31** was predominantly generated with no formation of the corresponding aziridine product (Fig. 3d) [59].

## Cyclopropanation

**Fig. 5:**
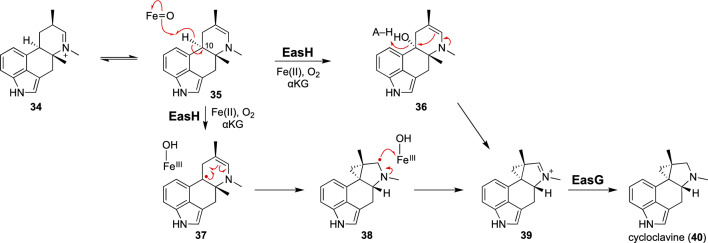
Cyclopropanation catalyzed by EasH in cycloclavine biosynthesis

Cycloclavine (**40**) is a cyclopropane ring-containing ergot alkaloid from the filamentous fungus, *Aspergillus japonicus* (Fig. 5) [60–63]. The biosynthetic gene cluster responsible for cycloclavine biosynthesis encodes seven enzymes [64]. Expression of the biosynthetic genes in *Saccharomyces cerevisiae* revealed that the cyclopropyl amine moiety is constructed during the last two steps of cycloclavine biosynthesis [65]. Namely, the αKG-dependent nonheme iron oxygenase EasH catalyzes the cyclopropanation of **34**/**35** to generate **39**, which is then reductively transformed to cycloclavine (**40**) by the NADPH-dependent reductase EasG. A related homologue of EasH from *Claviceps purpurea* catalyzes the C–O bond-forming cyclization of dihydroergotaman to dihydroergotamine [66]. Another homologue from an *Epichlo* sp. was also analyzed by heterologous gene expression, but its catalytic function remains unclear [67]. Two possible mechanisms for the EasH-catalyzed cyclopropanation are shown in Fig. 5 [65]. The substrate of EasH may exist as a mixture of the imine (**34**) and enamine (**35**) forms. An Fe(IV)-oxo species may abstract an H atom from C10 in **35**, followed by hydroxyl rebound to generate **36**. Then, C–C bond formation may occur with elimination of the newly installed hydroxyl group to produce **39**. This intramolecular substitution process is hypothesized to require acid–base chemistry. However, the X-ray crystal structure of EasH from *A. japonicus* suggested that the protein active site is constructed primarily by hydrophobic residues [68]. Therefore, a non-polar mechanism that is not assisted by polar active site residues has also been proposed (Fig. 5). The radical in **37** may intramolecularly add across the double bond of the enamine moiety to generate **38**, which is further oxidized into the imine product **39**. The mechanism involving the radical-based C–C bond formation step has also been suggested by QM/MM calculations [69]. These studies characterized EasH as the first example of cyclopropanation catalysis via αKG-dependent nonheme iron chemistry, although further examination will be necessary to test the hypothetical mechanisms.

**Fig. 6:**
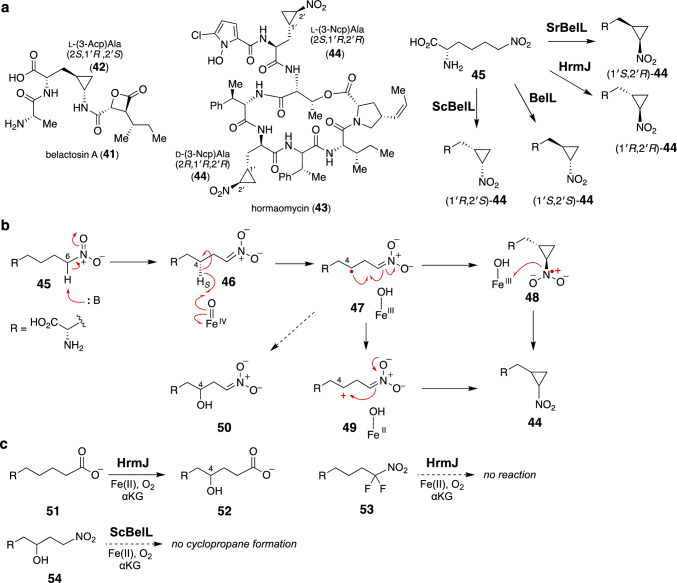
**a** Stereodivergent cyclopropanation catalyzed by nonheme iron enzymes. **b** Possible reactions mechanism for the cyclopropanation reaction. **c** Reactions of substrate analogues

The second examples of αKG-dependent nonheme iron cyclopropanases were discovered in the biosynthetic pathways of belactosin A (**41**) and hormaomycin (**43**) (Fig. 6a). Belactosin A (**41**) and hormaomycin (**43**) are distinct types of peptide natural products from Streptomycetes [70–74]. Belactosin A (**41**) has a β-lactone warhead conjugated with a (1′*R*,2′*S*)-3-(2-aminocyclopropyl)alanine (**42**, Acpa) residue. The structure of hormaomycin (**43**) features several unusual (amino)acid units, including 5-chloro-1-hydroxypyrrole-2-carboxylic acid, 3-propenylproline, and (1′*R*,2′*R*)-3-(2-nitrocyclopropyl)alanine (**44**, Ncpa) [75, 76]. (1′*R*,2′*S*)-Acpa (**42**) and (1′*R*,2′*R*)-Ncpa (**44**) are similar to each other, but possess different nitrogen substituent oxidation states and cyclopropane ring stereoconfigurations. The resemblance between Acpa (**42**) and Ncpa (**44**) implied that they share a common biosynthetic pathway, in which l-lysine serves as a precursor [77, 78]. The biosynthetic gene clusters of belactosin A (**41**) and hormaomycin (**43**) were independently discovered [79, 80]. Gene analysis suggested that the peptide structure of hormaomycin (**43**) is assembled by NRPSs, while the peptide bonds in belactosin A (**41**) are constructed by ATP-grasp ligases. Notably, the two biosynthetic gene clusters contain three pairs of the homologous genes *belK*-*hrmI*, *belL*-*hrmJ*, and *belM*-*hrmT*. Gene deletions in the belactosin A-producing strain *Streptomyces* sp. UCK14, combined with in vitro enzyme assays, indicated that the heme oxygenase-like diiron enzymes BelK and HrmI have the same catalytic function to oxidize l-lysine and generate l-6-nitronorleucine (**45**) [81–83]. Subsequently, the nonheme iron enzymes BelL and HrmJ similarly catalyze the dehydrogenative cyclization of the nitroalkane moiety of l-6-nitronorleucine (**45**) to generate Ncpa (**44**) [81, 82]. Consistent with the stereoconfigurations found in belactosin A (**41**) and hormaomycin (**43**), BelL produces (1′*S*,2′*S*)-Ncpa, while HrmJ produces (1′*R*,2′*R*)-Ncpa despite its moderate sequence identity (49% identity). The homologous genes *belM* and *hrmT* may encode diaminopimelate epimerases that could supply l-lysine as a precursor for Acpa (**42**) and Ncpa (**44**). It should also be noted that, in belactosin A (**41**) biosynthesis, the nitro group in (1′*S*,2′*S*)-Ncpa ((1′*S*,2′*S*)-**44**) is predicted to be reduced by the molybdenum-dependent reductase BelN [84].

A recent bioinformatic analysis of the *belL* gene revealed that many bacterial strains of *Actinomycetota* and *Proteobacteria* have *belL* gene homologues [85]. In vitro analysis of the l-6-nitronorleucine (**45**) reaction with purified BelL homologues indicated that they also produce the *cis* isomers (Fig. 6a). For example, the BelL homologue from *Streptomyces rimosus* subsp. *Paromomycinus* NBRC 15454 (SrBelL) generates (1′*S*,2′*R*)-Ncpa ((1′*S*,2′*R*)-**44**), while that from *Streptomyces cavourensis* NBRC 13026 (ScBelL) generates (1′*R*,2′*S*)-Ncpa ((1′*R*,2′*S*)-**44**). Therefore, this group of nonheme iron cyclopropanases produces one of the four possible diastereomers of Ncpa (**44**) with a remarkable stereocontrol mechanism.

Possible mechanisms for the cyclopropanation of l-6-nitronorleucine (**45**) are shown in Fig. 6b [81, 82, 85]. The α-carbon (C6) of the nitro group in l-6-nitronorleucine (**45**) may be deprotonated by a base in the active site to generate the nitronate intermediate **46**. Then, an Fe(IV)–oxo species abstracts an H atom from the C4 position to form the radical intermediate **47**. Several different pathways can be envisioned. The radical **47** may undergo radical addition across the double bond of the nitronate moiety to form the cyclopropane ring with the nitro anion radical **48**. Further oxidation of this species could afford the product Ncpa (**44**). An alternative mechanism involving the cation intermediate **49** should also be considered. It is also possible that the radical in **47** receives the hydroxyl group from the Fe(III) to form the C4–OH intermediate **50**, which might be cyclized into **44** via an intramolecular substitution reaction. It was suggested that the C6-deprotonation of l-6-nitronorleucine (**45**) could occur non-enzymatically at pH 7.5 [85]. Nevertheless, the addition of BelL to l-6-nitronorleucine (**45**) accelerated the rate of C6-deprotonation. The deprotonation is critical for the cyclization activity, because the fluorinated substrate analogue **53** that could not be deprotonated was not accepted by HrmJ, while the substrate analogue **51** containing a carboxylate moiety as a mimic of the nitro group in **45** was only hydroxylated, without the formation of the cyclized product (Fig. 6c). In addition, the enzymatic cyclization of l-6-nitronorleucine (**45**) was promoted under basic conditions (pH 9–11), consistent with the C6-deprotonation as a critical step.

Similar to other αKG-dependent nonheme iron enzymes, BelL and its homologues utilize an Fe(IV)-oxo species to abstract an H atom from the substrate or **46**, as confirmed by a transient kinetic analysis using stopped-flow absorption spectroscopy and Mössbauer spectroscopy [85]. The site of H abstraction from C4 was also determined to be the pro-*S*–H, based on the observed loss of the deuterium atom when (4*S*)-[4-^2^H]-**45** was employed as the substrate of BelL (Fig. 6b) [81, 85]. Notably, all other tested homologues of BelL similarly abstract pro-*S*–H, regardless of the diastereoselectivity of the reactions they catalyze. The radical **47** is likely cyclized directly into **44** via either a cation- or radical-based mechanism, since the synthetic 4-hydroxyl compound **54** was not converted to Ncpa (**44**), effectively excluding the possibility of the involvement of **50** (and **54**) as an on-pathway intermediate. DFT calculations suggested that the radical-based cyclization of **47** to **48** is energetically feasible, with an activation energy (Δ*G*^‡^) of 7.4 kcal mol^–1^. It is possible that the delocalization of the spin density over the three heteroatoms may contribute to the stabilization of **48**, thereby overcoming the inherent ring strain of the cyclopropane ring.

**Fig. 7:**
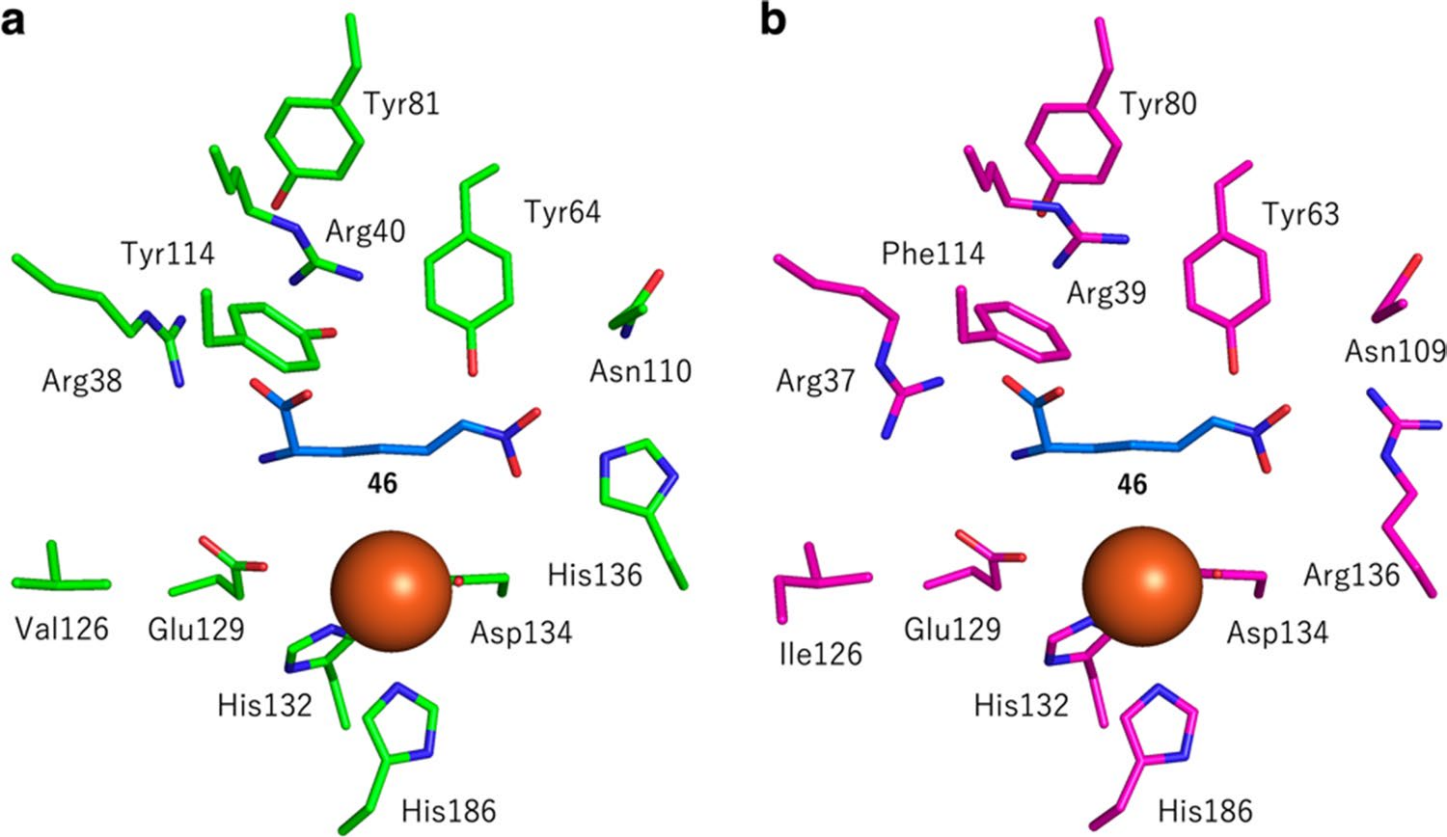
Comparison of the predicted structures of **a** BelL and **b** HrmJ docked with the nitronate form of the substrate **46** [85]

BelL and its homologues exhibit remarkable stereochemical control during the dehydrogenative cyclization of l-6-nitronorleucine (**45**) (Fig. 6a). The contributions of the active site residues toward the stereochemical outcomes are partially understood, based on structural analyses of this class of enzymes [85]. The X-ray crystal structure of ScBelL suggested that it resembles l-isoleucine dioxygenase (IDO) from *Bacillus thuringiensis*, consisting of a jelly roll fold with two anti-parallel β-sheets. Sequence alignments and comparisons of the homology models among the tested cyclopropanases revealed that many of the active site residues are conserved, while a few amino acid residues differ. For example, BelL has His at position 136, while the corresponding residue in HrmJ is substituted with Arg (Fig. 7). These residues are critical in controlling the product stereochemistry, because the BelL-H136R variant produced (1′*R*,2′*R*)-Ncpa ((1′*R*,2′*R*)-**44**) without forming the original product (1′*S*,2′*S*)-Ncpa ((1′*S*,2′*S*)-**44**). As expected, the HrmJ-R136H variant generated (1′*S*,2′*S*)-Ncpa ((1′*S*,2′*S*)-**44**), although the variant retained some activity to produce (1′*R*,2′*R*)-Ncpa ((1′*R*,2′*R*)-**44**). When the amino acid substitution was rationally designed based on sequence comparisons with homologues that produce *cis*-Ncpa (**44**), BelL was engineered to produce *cis*-Ncpa (**44**). However, the rational substitution of the active site residues did not necessarily lead to the desired changes of the product stereochemistry [85]. Thus, further investigations will be needed to clarify how these cyclopropanases control selectivity with only subtle differences in their primary sequences.

**Fig. 8:**
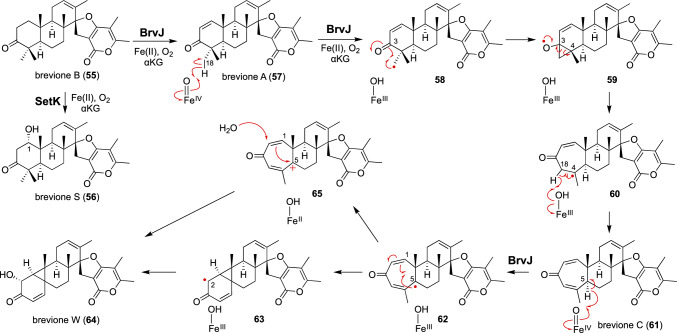
Reactions catalyzed by BrvJ and SetK in the biosynthesis of breviones

Another αKG-dependent nonheme iron cyclopropanase was recently characterized in the biosynthetic pathway of breviones [86] (Fig. 8). Breviones are meroterpenoid natural products from fungal strains such as *Penicillium brevicompactum* and *Penicillium bialowiezense* CBS 227.28 [87]. Brevione B (**55**) is biosynthesized via combined polyketide and terpenoid biosynthetic machineries and is proposed to be a common intermediate for setosusin and brevione E, a highly functionalized derivative of breviones. The biosynthetic gene clusters of setosusin and breviones encode two related nonheme iron enzymes, SetK and BrvJ, respectively [86, 88]. Heterologous expression revealed that SetK catalyzes brevione B (**55**) hydroxylation at the C1 position, while BrvJ performs oxidative ring expansion of the same intermediate to generate brevione C (**61**) via brevione A (**57**) [86]. BrvJ further converted brevione C (**61**) into the cyclopropane-containing brevione W (**64**) under the tested in vitro conditions. Structure-based mutagenesis of the active site residues (F159L/A227L/L238M) converted BrvJ into a C1-hydroxylase like SetK that generates brevione S (**56**).

The ring expansion of brevione A (**57**) may be initiated by H atom abstraction from C18 (Fig. 8) [86]. The resulting radical **58** rearranges to **60** through the O-centered radical with a cyclopropane ring (**59**), followed by H atom abstraction from C18 to produce brevione C (**61**). In the next catalytic cycle, an H atom from C18 in brevione C (**61**) may be abstracted to generate **52**, which undergoes radical addition onto the enone moiety to form the radical **63** with a cyclopropane ring. Finally, hydroxyl rebound with the Fe(III)-OH species may occur to produce **64**. Alternatively, the introduction of the hydroxy group at C2 may be effected via the carbocation intermediate **65**.

## Conclusions

Recent advancements in genome sequencing and bioinformatic technologies have enabled the discovery of nonheme iron-dependent cyclases producing epoxides, aziridines, and cyclopropanes, with H6H from the 1980s serving as an exception. Accumulated mechanistic knowledge suggests that, following C–H bond heterolytic cleavage, the key ring-closure step is likely achieved through either radical addition across an unsaturated bond or cation-mediated bond formation. However, unresolved enzymological questions remain to be answered. For example, it is not fully understood how these enzymes catalyze selective cyclization while avoiding hydroxylation, which is the typical outcome of αKG-dependent nonheme iron enzyme catalysis. Further mechanistic investigations will create new opportunities to develop practical biocatalysts for challenging cyclization reactions beyond the capabilities of current organic synthesis technology.
